# Respiratory Tract Infections and the Role of Biologically Active Polysaccharides in Their Management and Prevention

**DOI:** 10.3390/nu9070779

**Published:** 2017-07-20

**Authors:** Milos Jesenak, Ingrid Urbancikova, Peter Banovcin

**Affiliations:** 1 Department of Pediatrics, Comenius University in Bratislava, Jessenius Faculty of Medicine in Martin, University Teaching Hospital, Kollarova 2, 036 59 Martin, Slovakia; galandovedni@gmail.com; 2 Department of Pediatrics, P.J. Safarik University, Faculty of Medicine, Children’s Faculty Hospital, Trieda SNP 1, 040 11 Kosice, Slovakia

**Keywords:** β-glucans, biologically active polysaccharides, immunomodulation, recurrent respiratory tract infections, prevention

## Abstract

Respiratory tract infections (RTIs) are the most common form of infections in every age category. Recurrent respiratory tract infections (RRTIs), a specific form of RTIs, represent a typical and common problem associated with early childhood, causing high indirect and direct costs on the healthcare system. They are usually the consequence of immature immunity in children and high exposure to various respiratory pathogens. Their rational management should aim at excluding other severe chronic diseases associated with increased morbidity (e.g., primary immunodeficiency syndromes, cystic fibrosis, and ciliary dyskinesia) and at supporting maturity of the mucosal immune system. However, RRTIs can also be observed in adults (e.g., during exhausting and stressful periods, chronic inflammatory diseases, secondary immunodeficiencies, or in elite athletes) and require greater attention. Biologically active polysaccharides (e.g., β-glucans) are one of the most studied natural immunomodulators with a pluripotent mode of action and biological activity. According to many studies, they possess immunomodulatory, anti-inflammatory, and anti-infectious activities and therefore could be suggested as an effective part of treating and preventing RTIs. Based on published studies, the application of β-glucans was proven as a possible therapeutic and preventive approach in managing and preventing recurrent respiratory tract infections in children (especially β-glucans from *Pleurotus ostreatus*), adults (mostly the studies with yeast-derived β-glucans), and in elite athletes (studies with β-glucans from *Pleurotus ostreatus* or yeast).

## 1. Recurrent Respiratory Tract Infections and Their Management

Respiratory tract infections (RTIs) represent the most common form of infection at every age. Studies have shown that with increasing age, the incidence of respiratory infection declines. However, in specific age (preschool age) or subject groups (e.g., patients with chronic diseases, immunosuppressive therapy, athletes), the frequency of RTIs is so high that diagnosis of recurrent respiratory tract infections (RRTIs) ought to be discussed. Several possible definitions of RRTIs are in the literature, but the most commonly used are those stated by de Martino et al. (2007). Patients with RRTIs should fulfill at least one of the following criteria: ≥6 respiratory infections per year; ≥1 respiratory infection per month involving the upper airways from September to April; or ≥3 respiratory infections per year involving the lower airways [1]. RRTIs are a specific type of respiratory infection with higher frequency compared to the acceptable and expectable number of RTIs for a particular age group (so-called physiological morbidity). Correct diagnosis of RRTIs can be established after excluding some severe and chronic conditions associated with RRTIs: cystic fibrosis, primary and secondary immunodeficiency syndromes, primary and secondary ciliary dyskinesia syndromes, or congenital anomalies of the respiratory tract. Also, many factors may contribute to higher respiratory morbidity: older siblings of close age and larger families (with increased possibility to get in contact with RTIs), day-care center attendance, prematurity, shortened breast feeding and malnutrition, environmental factors (indoor and outdoor pollution, passive smoking), allergic inflammation (especially in children of risk with allergic, first-degree relatives), chronic focal infections (e.g., adenoid hypertrophy, chronic tonsillitis, sinusitis) or gastroesophageal reflux. In preschoolers, immature mucosal immunity is another important factor [2,3,4]. 

The majority of patients with RRTIs do not have any recognizable immunodeficiencies or other pathologies, but some of these subjects may have mild and non-specific deviations in selected immune parameters that are an expression of immature immunity, or transient immune function decline after certain events (e.g., extreme physical activities or post-infectious, stressful, and exhausting periods) [1]. Immunomodulation represents one of the possible and accepted approaches in managing and preventing RRTIs at every age or patient category. Immunomodulation is characterized as a preventive and therapeutic intervention into immune system activity aimed at correcting deviated immune functions. It can either support declined and suppressed immune parameters or normalize the increased and over-acting functions. Many natural and synthetic compounds and medications have immunomodulatory activities, many of them based on traditional medicine. However, only few of them also possess a scientific basis.

## 2. Biologically Active Polysaccharides as Biological Response Modifiers

Biologically active polysaccharides (BAPs) are one of the most studied natural immunomodulators and due to their confirmed complex mode of action, they can be named as biological responses modifiers. The most important BAPs are β-glucans, which are a heterogeneous group of natural polysaccharides, comprising d-glucose monomers linked by β-glycosidic bonds. Several sources of β-glucans exist: fungi, yeasts, bacteria, algae, and various plants. Studies have shown that immunomodulating potential differs between β-glucans due to their origin, purity, structure, branching level, solubility, and molecular conformation [5,6,7,8,9]. Studies have also shown that the β-glucans can yield similar activities when used in both injectable and oral forms [5].

Studies have shown that β-glucans possess many biological activities. Those receptors on immune and non-immune cell surfaces (e.g., fibroblasts, keratinocytes) which are important in mediation of β-glucan activities include: dectin-1, complement receptor 3, toll-like receptors (TLR), and others [10,11]. Dectin-1 represents probably the most important receptor mediating the biological effects of β-glucans. It is expressed especially on the cells of non-specific immunity, e.g., macrophages, neutrophils, and dendritic cells. It closely collaborates with TLR 2 and 6 (TLR-2/6). Its activation is linked to the several intracellular pathways (e.g., nuclear factor κB or signaling adaptor protein CARD9), which lead to the release of various cytokines. TLR-4 is probably another essential receptor involved in the activation and maturation of dendritic cells after the recognition of β-glucans [11]. Since insoluble β-glucans are not absorbed into the blood, there are several theories or hypotheses trying to explain their mode of action. One possibility is represented by the direct interaction with immune cells in Peyer’s patches in small intestine with subsequent immune cell activation [12]. Another possible mechanism could be the ingestion of β-glucans by macrophages followed by the release of fragments into the intercellular microenvironment and activation of other cellular populations. β-glucans can activate innate as well as adaptive immune mechanisms and cells, stimulate the activity of neutrophils and macrophages via surface receptors, make the phagocytosis more effective, support the functions of NK cells, modulate the functions of antigen-presenting cells (e.g., Langerhans cells) and promote antigen presentation, influence the production of cytokines and chemokines, create conditions supporting Th1 lymphocytes, and modulate antibody production [3,13,14,15,16,17]. 

β-glucans are characterized by pluripotent biological properties which can be useful in managing various immune-mediated conditions and infectious diseases, e.g., RRTIs. In terms of managing and preventing RTIs, a number of published reports exist with children or adult subjects in the literature that studied the β-glucans from oyster mushrooms (*Pleurotus ostreatus*), baker’s yeast (*Saccharomyces cerevisiae*) and oats (Table 1). In our study we aimed to analyze the possible effect of commonly used insoluble β-glucans in the treatment and prevention of RTIs in children, adults and in athletes. Original articles have been selected for analysis among those published in PubMed and Scopus referenced journals using the following keywords: “respiratory infections”, “respiratory tract infections”, “recurrent respiratory tract infections”, “treatment”, “prevention”, “β-glucans”, “glucans”. For the final analysis, only the studies evaluating the effects of orally applied β-glucans on various clinical and laboratory parameters in human subjects with respiratory tract infections were selected. 

## 3. β-Glucans Isolated from *Pleurotus Ostreatus* and Respiratory Tract Infections

The first study analyzing the efficacy and safety of syrup containing a patented complex of biologically active polysaccharides isolated from *Pleurotus ostreatus* was performed in the Czech and Slovak Republics in a group of 215 children, mostly preschoolers. Children were administered a syrup containing pleuran—insoluble β-glucans from *Pleurotus ostreatus*—every morning on an empty stomach for 3 months starting at the beginning of Autumn. A positive therapeutic response (more than 50% reduction of RRTI frequency) was observed in 71.2% of the studied children and the total number of respiratory tract infections declined from 8.9 episodes of RTIs/year to 3.6 episodes/year compared to the previous treatment period (*p* < 0.001) [18]. 

In another, open-label study from Spain, the effect of the same product on frequency of RTIs and other selected parameters was studied in a group of 151 children. The children were administered the medication for 3 months and were followed-up for 3 additional months. Active treatment decreased the number of the RRTIs from 8.88 ± 3.35 episodes in the previous year to 4.27 ± 2.21 episodes in the study year (*p* < 0.001). Furthermore, the incidence and number of episodes of each type of respiratory tract infection (otitis media, common cold, tonsillopharyngitis, laryngitis, bronchitis, and pneumonia) were significantly reduced. Application of the syrup with pleuran also reduced the number of emergency department visits, use of symptomatic pharmacotherapy, and missed days from kindergarten or school compared to the previous year before treatment. The product showed good or very good tolerability in 90.7% of children and a significant improvement of clinical status was reported by 85.7% of parents [19]. A study of the same design and product was later performed in another group of 194 children in Poland. Generally, supplementation of syrup with pleuran (Imunoglukan P4H^®^ syrup) significantly decreased the total number of RTIs during treatment and the follow-up period compared to the same period of the previous year (4.18 ± 2.132 vs. 8.71 ± 1.89, *p* < 0.001). The syrup demonstrated a significant capacity to prevent and decrease the number of various forms of RTIs (otitis media, laryngitis, bronchitis and common cold) and positively influenced the number of days off from kindergarten or school. As in the previous trials, the product was well tolerated and no serious or adverse events were observed [20]. 

The positive and preventive effects of pleuran supplementation on respiratory morbidity were also confirmed in two double-blind, placebo-controlled, multicenter randomized trials (DBPCRT). The only published DBPCRT analyzing the preventive effect of β-glucan on RRTIs was performed in a population of 175 children aged 5.65 years. The subjects were randomized into two different treatment groups receiving either syrup containing pleuran and vitamin C or an active placebo—syrup with vitamin C. The children were administered the medication on an empty stomach for 6 months (starting in August to October) and were followed-up for 6 additional months. In the active group, 36% of children did not have any respiratory infections during the treatment period compared to 21% in the placebo group (*p* < 0.05). Active treatment also significantly decreased the frequency of flu and flu-like diseases (*p* < 0.05) as well as the frequency of lower respiratory tract infection (*p* < 0.05). Based on the laboratory results, active treatment showed potential immunomodulatory effects on humoral immunity and supported natural maturation of antibody production. There were no signs of overstimulation in the cellular part of the immune system. Vitamin C was used as an active placebo to demonstrate that the observed laboratory or clinical effects can be attributed to the active substance—pleuran—not to vitamin C, which is also contained in studied Imunoglukan P4H^®^ syrup [3]. Another DBPCRT investigated the preventive effect of pleuran on infectious and non-infectious complications in adult patients with Crohn’s disease. All the patients were treated with biological therapy (TNF-α blockers) and were enrolled during the clinical remission of the gastrointestinal disease. These patients usually suffer from recurrent respiratory tract infections due to a complex immunodeficiency of combined origin (immunosuppressant therapy, chronic inflammation, immune dysregulation), each emerging infection having the capacity to alter the disease’s stability. Actively treated patients showed decreased frequency of accompanying diseases and emerging infections compared to the placebo arm of the study. Moreover, pleuran was safe and did not worsen the clinical course of Crohn’s diseases [21].

## 4. Yeast and Oat β-Glucans and Respiratory Tract Infections

Other traditionally used β-glucans are isolated from yeast or cereals (mostly from oats). Studies show that β-glucans from various sources (mushrooms, yeast, and cereals) and within one group (e.g., β-glucans from yeasts) could have different immunomodulatory potential and varying clinical effects [7]. 

Several clinical studies and reports analyze the effects of yeast and cereal β-glucans on RTIs or selected immune parameters. Most of these studies were performed in adult populations. Auinger et al. (2013) investigated the effect of baker’s yeast (*Saccharomyces cerevisiae*) on the number of common cold episodes in 162 healthy subjects who were randomized into either active or placebo arms. Supplementation of insoluble yeast β-glucan reduced the number of symptomatic cold infections by 25% compared to placebo (*p* = 0.041) with improvement of sleep difficulties caused by cold episodes (*p* = 0.028). The efficacy of the preparation was positively rated by both patients and physicians [22]. Another authors’ group examined the effects of yeast β-glucans in different clinical trials. In a group of 77 stressed, adult women (with moderate level of psychological stress), subjects treated with yeast β-glucan reported fewer upper respiratory symptoms compared to placebo (*p* < 0.05). Active treatment was associated with better overall well-being and superior mental and physical energy levels (*p* < 0.05) [23]. In another study by the same authors, 150 moderately to highly stressed subjects (45 men, 105 women) took either yeast β-glucan (250 mg or 500 mg) or placebo with the aim of influencing the onset of respiratory tract infection and well-being. Two dosing regimens decreased upper respiratory tract infection symptoms (*p* < 0.05) and improved overall health and vigor (*p* < 0.05) with decreased tension and fatigue (*p* < 0.05) compared to placebo. There were no statistically significant differences between two dosing schedules of β-glucan [24]. 

Some studies have shown no significant differences in the incidence of respiratory tract infection between treatment with β-glucan or placebo. The study with yeast β-glucan in 40 healthy adults did not show any differences in the incidence of symptomatic respiratory tract infection among the study groups (β-glucan versus placebo). However, none of the actively treated subjects missed days from work or school (*p* = 0.026). Moreover, the application of β-glucan improved quality of life (*p* = 0.042) and decreased average fever scores (*p* = 0.042) compared to placebo-treated subjects [25]. Another study also did not observe any differences in the incidence of common cold episodes compared to placebo. However, in the β-glucan-treated patients, a higher number of subjects without incidence of common cold was seen (*p* = 0.019). During the period with the highest incidence of infections, active treatment led to significantly less infection and reduced typical common cold symptoms (the symptoms were less pronounced and subsided faster) (*p* = 0.020) [26]. Fuller at al. (2012) could not discover a statistically significant difference in the number of days with upper respiratory tract infection symptoms when comparing the use of 250 mg of yeast β-glucan to the rice flour-based placebo. The only significant outcome of their study was improved ability to “breathe easily” in the active arm (*p* = 0.049). Additionally, no significant changes in chemokines or cytokines production between the two groups was found [27]. 

A couple of papers with yeast-derived β-glucans in the management of children with chronic respiratory problems were published by the group of Vetvicka & Richter [28,29,30,31,32,33]. In the similar cohorts of the children, they evaluated the effect of yeast-derived insoluble β-glucan on different salivary parameters and clinical characteristics. In the first study, they examined salivary inflammatory markers in 40 children with chronic respiratory diseases (recurrent respiratory tract infections, chronic bronchitis, bronchial asthma, respiratory allergies). An oral application over 4 weeks of insoluble yeast β-glucan decreased the concentration of albumin and increased the levels of lysozyme in saliva among the actively treated children, whereas in placebo group, no significant changes were recorded. The authors reported an improvement in general condition regarding chronic respiratory diseases, however, more detailed information cannot be found in this publication [28]. In other two studies they showed positive effect of orally applied β-glucan on the concentration of salivary immunoglobulin A, G and M levels [29] or inflammatory markers (lysozyme, calprotectin, albumin and CRP) in saliva [30]. Some of the results were inconsistent and differed between the publications (e.g., changes in salivary concentration of lysozyme) [28,30]. Concentration of cotinine (a marker of passive smoking exposure) and cortisol decreased in saliva after a 4-week-application of yeast β-glucan compared to placebo. Therefore authors suggested that β-glucan is able to reduce the negative environmental effects on children with chronic respiratory problems [31]. The possible preventive effect of β-glucan in children under physical stress was studied in another two trials of this authors’ group. They found significant improvements in physical endurance and exhaled nitric oxide in glucan-treated children [32]. They observed a stabilization of the IgA levels in saliva after a 6-min walking test compared to placebo [33]. Conversely, levels of exhaled nitric oxide decreased in both actively or placebo treated children [32,33]. The preventive effect of β-glucan on RTIs was not evaluated in these studies. 

Another interesting approach was studied by Li et al. (2014) in a double-blind, randomized, controlled prospective trial. They analyzed the possible effect of the follow-up formula containing docosahexaenoic acid (DHA), prebiotics PDX/GOS (polydextrose and galacto-oligosaccharides 1:1 ratio), and 8.7 mg yeast β-glucan per dose on respiratory infection compared to children administered an unfortified, cow’s milk-based beverage for 28 weeks. Children administered the modified follow-up formula (FUF) had fewer episodes and shorter duration of acute respiratory tract infections, less antibiotic use, and fewer missed days of day-care. The FUF group also had a higher blood concentration of interleukin 10 and white blood cell count at the end of the study. This strategy could be considered a promising tool for improving and supporting maturity of the immune system during early life via feeding. However, whether the observed effect was strictly attributable to only β-glucan or also to the prebiotics or DHA cannot be determined [34].

## 5. β-Glucans, Recurrent Respiratory Tract Infections, and Sports Medicine

Sports activity has many beneficial health effects at both a physical and mental level. It was shown that short term physical activity has immune activating effects. Conversely, prolonged and exhausting physical activity causes numerous negative changes to immunity. These changes are usually transient, but in the absence of sufficient resting time, immunosuppression becomes more profound and the development of secondary immunodeficiency can be observed. This leads to an increased risk of RTIs with a general negative impact on sports performance. It is possible that supplementing various immunomodulators could minimize post-exercise immunosuppression, improve immune functions, and decrease the rate and severity of RTIs [35]. The characteristics and outcomes of available studies performed with different β-glucans in athletes are summarized in Table 2.

Some studies have only analyzed the effect of different β-glucans on selected laboratory and immune parameters. In a group of 60 recreationally active men and women, 10 days of supplementing with baker’s yeast β-glucan resulted in an increase of total (CD14^+^) and pro-inflammatory monocyte (CD14^+^CD16^+^) concentrations. Furthermore, β-glucan boosted lipopolysaccharide-stimulated production of IL-2, IL-4, IL-5, and IFN-γ before and after exercise. Plasma concentration of IL-4, IL-5, and IFN-γ were also greater 2 h after exercise in the β-glucan group compared to the placebo group. Therefore, it can be suggested that β-glucans may modulate immune response and immune reactivity following strenuous exercise [36]. Bobovcak et al. (2010) confirmed that supplementing with pleuran prevented the decline of natural killer cell numbers and activity after the recovery period in elite athletes compared to placebo [37]. 

Various trials have shown that β-glucan supplementation could decrease RTIs in athletes. Supplementation with insoluble pleuran from *Pleurotus ostreatus* significantly decreased the incidence of upper respiratory tract infection compared to placebo in elite athletes (*p* < 0.001). Active treatment also increased natural killer cell numbers and prevented the decline of phagocytic functions after the physical exertion [38]. In a study with 75 marathon runners, yeast β-glucan was administered for 4 weeks and significantly decreased symptoms of upper respiratory tract infection, confusion, fatigue, anger, and tension, improving overall health and increasing vigor [39]. Similarly, McFarlin et al. (2013) reported a 37% reduction in the number of cold/flu symptom days post-marathon compared to placebo. Interestingly, 2 hours after exercise yeast β-glucan caused a 32% increase in salivary IgA compared to placebo [40]. Only one trial was performed with oat-derived β-glucan. Its application did not prevent or reduce post-exercise-induced immune changes or incidence of upper respiratory tract infection during the treatment and follow-up period [41].

## 6. Discussion

β-glucans represent a promising group of immunomodulatory substances with pluripotent biological activities and favorable safety profile. Up until now, several mechanisms of action have been supposed and at least partially confirmed, especially through the laboratory and animal studies. However, the exact mechanisms of biological effects in humans are still under investigation. The use of β-glucans in the management and prevention of RTIs have been evaluated in several studies with different patients populations (children, adults, stressed individuals, athletes). Clinical trials showed a potential role of β-glucans in modulation of mucosal and systemic immunity with positive effect on selected inflammatory markers. β-glucans also yielded a positive effect on the parameter of systemic and mucosal humoral immunity what precludes their potential in the management of RTIs. Moreover, an improvement of health status and general well-being was consistently reported. The preventive potential of β-glucans in RRTIs was confirmed especially for insoluble β-glucans isolated from *Pleurotus ostreatus* and until now, only one DBPCRT, which showed a preventive effect in RRTIs in children, was published. The studies with yeast β-glucans reported especially some effects on the incidence of upper respiratory symptoms, but clear preventive effect was not observed. In athletes, the prevention of post-exercise immune suppression and decreased incidence of RTIs was also confirmed. 

On the other hand, the published studies had several weaknesses which should be resolved and addressed in the further clinical trials and research. The number of the involved subjects was small in several studies and high inconsistency was noticed in the selection criteria of the patients. Heterogeneity of the studied cohorts caused inconsistent results found in some studies. Detailed information about the particular forms of RTIs treated and prevented by β-glucans is in general missing in the publications. Up to now, an optimal dose, duration and timing of the β-glucans’ application has not been clearly defined. Moreover, the applied β-glucans were not characterized in several studies and the purity of the active substance was not reported. 

More studies analyzing the preventive effect of β-glucans in the management of RRTIs are needed to confirm the existing data. For the future, the estimation of the optimal effective dose and the duration of the application of β-glucans from particular source should be evaluated. Another important issue which should be resolved is the standardization of the production and extraction with achieving the highest purity of the active substance from the natural β-glucans’ sources. Studies could also focus on the possible combinations of the β-glucans with another immune active substances. Recently, intranasal application of β-glucan in combination with resveratrol showed another promising approach for the prevention of upper respiratory tract infections. In a group of 82 children with RRTIs, intranasal application of the mixture of carboxymethyl-β-glucan and resveratrol was able to reduce the number of days with nasal obstruction, rhinorrhea, sneezing, cough, fever, medication use, medical visits and school absence compared to saline isotonic solution. Therefore, this mode of β-glucans’ application should be addressed in the further studies [42].

## 7. Conclusions

Respiratory tract infections represent an important health-care problem and their rational management and effective prevention could have many direct and indirect benefits. Recurrent respiratory tract infections are a special form of RTI typical in children and some specific patient groups. RRTIs have high direct and indirect economic costs and increasing antibiotic resistance is today a serious and emerging problem. Immunomodulation, therefore, represents an interesting approach how to decrease the use of antibiotics and alleviate the economic impact of RTIs. Based on published studies, evidence supports the preventive use of β-glucans in managing RRTIs. Whereas in children, especially β-glucans from *Pleurotus ostreatus* have proven to be effective and safe, most studies performed with adults were especially with yeast-derived β-glucans. Some data demonstrates the efficacy of β-glucans in preventing RTIs in elite athletes. Preventive application of β-glucans may decrease the frequency of various forms of respiratory tract infection, support protective immune mechanisms, and possibly yield other beneficial effects (increased well-being, decreased missed days from school or work, decreased use of other symptomatic or antibiotic therapy). 

## Figures and Tables

**Table 1:** Effect of β-glucans of different origin on the respiratory tract infections and selected parameters in children and adults.

No.	Country of Study	Study Population	Age	Study Design	Main Outcomes	β-Glucan Type (Dose)	Duration of Treatment	Reference
**1**	Czech and Slovak Republic	215 children with RRTIs	4.7 years	OLS	↓ frequency of RRTIs (positive therapeutic response—≥50% reduction of RRTI frequency—in 71.2% of children (*p* < 0.001)	Pleuran—insoluble β-glucan from *Pleurotus ostreatus* (10 mg/10 kg of body weight)	3 months (& 3 months follow-up)	Jesenak et al., 2010 [18]
**2**	Spain	151 children with RRTIs	3.0 years	OLS	↓ frequency of RRTIs (*p* < 0.001) ↓ number of otitis media (*p* < 0.001), common cold (*p* < 0.001), tonsillopharyngitis (*p* < 0.001), laryngitis (*p* < 0.001), bronchitis (*p* < 0.001), pneumonia (*p* < 0.05) ↓ number of emergency visits due to respiratory infections (*p* < 0.001) ↓ number of days-off from kindergarten or school (*p* < 0.05) ↓ use of symptomatic therapy (*p* < 0.05)	Pleuran—insoluble β-glucan from *Pleurotus ostreatus* (10 mg/10 kg of body weight)	3 months (& 3 months follow-up)	Sapena Grau et al., 2015 [19]
**3**	Poland	194 children with RRTIs	3.7 years	OLS	↓ frequency of RRTIs (*p* < 0.001) ↓ number of otitis media (*p* < 0.01), laryngitis (*p* < 0.01), bronchitis (*p* < 0.01), common cold (*p* < 0.01) ↓ number of days-off from kindergarten or school (*p* < 0.01)	Pleuran—insoluble β-glucan from *Pleurotus ostreatus* (10 mg/10 kg of body weight)	3 months (& 3 months follow-up)	Pasnik et al., 2017 [20]
**4**	Czech and Slovak Republic	175 children with RRTIs	5.6 years	DBPCRT	↓ frequency of RRTIs (*p* < 0.05) ↑ number of healthy children (*p* < 0.05) ↓ number of flu and flu-like diseases (*p* < 0.05) ↓ number of lower respiratory tract infections (*p* < 0.05) immunomodulating effects on antibody production immunomodulating effects on cellular immunity	Pleuran—insoluble β-glucan from *Pleurotus ostreatus* (10 mg/10 kg of body weight)	6 months (& 6 months follow-up)	Jesenak et al., 2013 [3]
**5**	Slovak Republic	53 adult patients with Crohn‘s disease	37.0 years	DBPCRT	↓ frequency of accompanying diseases (respiratory tract infections, herpes simplex infections, oral thrush) (*p* = 0.019) Ø effect of Crohn’s diseases activity	Pleuran—insoluble β-glucan from *Pleurotus ostreatus* (100 mg/day)	12 months	Batovsky et al., 2015 [21]
**6**	Germany	162 healthy adults	43.2 years	DBPCRT	↓ number of symptomatic cold episodes (*p* = 0.041) ↓ sleep difficulties caused by cold episodes (*p* < 0.028)	Insoluble yeast β-glucan (900 mg/day)	4 months	Auinger et al., 2013 [22]
**7**	U.S.A.	77 stressed adult women	38.0 years	DBPCRT	↓ upper respiratory symptoms (*p* < 0.05) ↑ overall well-being and superior mental/physical energy levels (*p* < 0.05)	Insoluble yeast β-glucan (250 mg/day)	3 months	Talbott et al., 2012 [23]
**8**	U.S.A.	150 moderately to highly-stressed adults	39.0 years	DBPCRT	↓ upper respiratory tract infection symptoms (*p* < 0.05) ↑ overall well-being and vigor (*p* < 0.05) ↓ fatigue and tension (*p* < 0.05)	Insoluble yeast β-glucan (250 or 500 mg/day)	1 month	Talbott et al., 2010 [24]
**9**	U.S.A.	40 healthy adults	30.3 years	DBPCRT	Ø differences in the incidence of symptomatic respiratory tract infection ↓ number of missed day of scholld or work per cold (*p* = 0.026) ↑ quality of life in active group (*p* = 0.042) ↓ average fever score (*p* = 0.042)	Insoluble yeast β-glucan (500 mg/day)	3 months	Feldman et al., 2009 [25]
**10**	Germany	94 healthy adults	45.6 years	DBPCRT	Ø differences in the incidence of common cold ↑ subjects without incidence of common cold compares to placebo (*p* = 0.019) ↓ number of infections during the most intense season for infection (*p* = 0.02) ↓ of typical common cold symptoms: sore throat and/or difficulty swallowing (*p* = 0.034), hoarseness and/or cough (*p* < 0.001), runny nose (*p* < 0.001)	Insoluble yeast β-glucan (450 mg/day)	7 months	Graubaum et al., 2012 [26]
**11**	United Kingdom	97 healthy adults	21.0 years	DBPCRT	Ø effect on the incidence of respiratory tract infection ↑ ability to “breathe easily” (*p* = 0.049) Ø effect on chemokines and cytokines production	Insoluble yeast β-glucan (250 mg/day)	3 months	Fuller et al., 2012 [27]
**12**	Czech Republic	40 children with chronic respiratory problems	10.7 years	DBPCRT	Improvement of mucosal immunity: ↑ lysozyme (*p* < 0.05), ↓ albumin (*p* < 0.05) Improvement in general disease condition	Insoluble yeast β-glucan (100 mg/day)	1 month	Vetvicka et al., 2013 [28]
**13**	Czech Republic	40 children with chronic respiratory problems	10.7 years	DBPCRT	↑ of salivary immunoglobulins (IgG, IgA, IgM) (*p* < 0.05)	Insoluble yeast β-glucan (100 mg/day)	1 month	Vetvicka et al., 2013 [29]
**14**	Czech Republic	60 children with chronic respiratory problems	9.7 years	DBPCRT	↓ of salivary lysozyme (*p* < 0.05), calprotectin (*p* = 0.015), albumin (*p* < 0.05)	Insoluble yeast β-glucan (100 mg/day)	1 month	Richter et al., 2014 [30]
**15**	Czech Republic	56 children with chronic respiratory problems	9.7 years	DBPCRT	↓ of salivary cotinine (*p* < 0.05) and cortisol levels (*p* < 0.05) ↑ of physical endurance (*p* < 0.05)	Insoluble yeast β-glucan (100 mg/day)	1 month	Richter et al., 2014 [31]
**16**	Czech Republic	40 children with chronic respiratory problems	10.9 years	DBPCRT	↑ of physical endurance (*p* < 0.05)	Insoluble yeast β-glucan (100 mg/day)	1 month	Vetvicka et al., 2013 [32]
**17**	Czech Republic	77 children with chronic respiratory problems	10.3 years	DBPCRT	Stabilization of the salivary IgA levels	Insoluble yeast β-glucan (100 mg/day)	1 month	Richter et al., 2015 [33]
**18**	U.S.A.	264 healthy children	3.5 years	DBPCRT	↓ number and duration of acute respiratory infections (*p* = 0.007) ↓ antibiotic use (*p* = 0.01) Immunomodulatory and anti-inflammatory effects	Insoluble yeast β-glucan (26.1 mg/day)	7 months	Li et al., 2014 [34]

DBPCRT—double-blind, placebo-controlled, randomized trial; OLS—open-label study; RRTIs—recurrent respiratory tract infections; ↑—increased/improved, ↓ decreased/worsened, Ø—no effect.

**Table 2:** Effect of β-glucans of different origin on respiratory tract infections and laboratory parameters in athletes.

No.	Country	Study Population	Age	Study Design	Main Outcomes	β-Glucan Type (Dose)	Duration of Treatment	Reference
**1**	U.S.A.	60 recreationally active adults	22.5 years	DBPCRT	↑ potential of blood leukocytes to produce IL-2, IL-4, IL-5, IFN-γ (*p* < 0.05) Effect on respiratory morbidity not studied	Insoluble yeast β-glucan (100 mg/day)	20 days (cross-over after 10 days)	Carpenter et al., 2013 [36]
**2**	Slovak Republic	20 elite athletes	23.3 years	DBPCRT	Prevention of decline in natural killer cell numbers and activity (*p* < 0.001) Effect on respiratory morbidity not studied	Pleuran—insoluble β-glucan from *Pleurotus ostreatus* (100 mg/day)	2 months	Bobovcak et al., 2010 [37]
**3**	Slovak Republic	50 elite athletes	23.6 years	DBPCRT	↓ incidence of upper respiratory tract infections (*p* < 0.001) ↑ number of natural killer cells (*p* < 0.001) Prevention of decline of phagocytic functions (*p* < 0.001)	Pleuran—insoluble β-glucan from *Pleurotus ostreatus* (200 mg/day)	3 months (& 3 months follow-up)	Bergendiova et al., 2010 [38]
**4**	U.S.A.	75 marathon runners	36.0 years	DBPCRT	↓ number of upper respiratory tract infection symptoms (*p* < 0.05) ↑ overall health and vigor (*p* < 0.05) ↓ confusion, fatigue, tension, and anger (*p* < 0.05)	Insoluble yeast β-glucan (250 or 500 mg/day)	1 month	Talbott et al., 2009 [39]
**5**	U.S.A.	182 marathon runners	34.0 years	DBPCRT	↓ number of cold/flu symptom days (*p* = 0.026) ↑ salivary IgA after exercise (*p* < 0.05)	Insoluble yeast β-glucan (250 mg/day)	1 month	McFarlin et al., 2013 [40]
**6**	U.S.A.	36 trained male cyclists		DBPCRT	Ø effect on incidence of upper respiratory tract infections Ø effect on exercise-induced immune changes	Insoluble oat β-glucan (5.6 g/day)	2 weeks (+ & weeks follow-up)	Nieman et al., 2008 [41]

DBPCRT—double-blind, placebo-controlled, randomized trial; IFN—interferon; IL—interleukin; RRTIs—recurrent respiratory tract infections; ↑—increased/improved, ↓ decreased/worsened, Ø—no effect.
